# Development and external validation of prediction risk scores (STRISK and NOFA) to predict immediate surgical need in adhesive small bowel obstruction: an observational prospective multicentre study

**DOI:** 10.1093/bjs/znaf025

**Published:** 2025-03-19

**Authors:** Panu Räty, Akseli Bonsdorff, Helka Parviainen, Eila Lantto, Thomas Hackenberg, Hanna Lampela, Taina Nykänen, Ilana Lyytinen, Panu Mentula, Ville Sallinen

**Affiliations:** Gastroenterological Surgery, Helsinki University Hospital and University of Helsinki, Helsinki, Finland; Gastroenterological Surgery, Helsinki University Hospital and University of Helsinki, Helsinki, Finland; HUS Medical Imaging Center, Radiology, Helsinki University Hospital and University of Helsinki, Helsinki, Finland; HUS Medical Imaging Center, Radiology, Helsinki University Hospital and University of Helsinki, Helsinki, Finland; Department of Surgery, Hyvinkää Hospital, Hyvinkää, Finland; Gastroenterological Surgery, Helsinki University Hospital and University of Helsinki, Helsinki, Finland; Department of Surgery, Hyvinkää Hospital, Hyvinkää, Finland; Department of Surgery, Kanta-Häme Central Hospital, Hämeenlinna, Finland; Gastroenterological Surgery, Helsinki University Hospital and University of Helsinki, Helsinki, Finland; Gastroenterological Surgery, Helsinki University Hospital and University of Helsinki, Helsinki, Finland; Department of Transplantation and Liver Surgery, Helsinki University Hospital and University of Helsinki, Helsinki, Finland

## Abstract

**Background:**

Adhesive small bowel obstruction (SBO) is a common cause of emergency admission. Identification of patients at high risk of strangulation or failure of non-operative treatment is difficult. In this multicentre prospective observational study, prediction models for strangulation and non-operative treatment failure in adhesive SBO were developed.

**Method:**

This study was carried out in three Finnish hospitals between June 2014 to May 2022. Patients with CT-confirmed adhesive SBO and prospective case report forms were included. The main outcomes were strangulation defined by operative finding of any intestinal ischaemia and failure of non-operative treatment within 30 days from admission. The model was developed using binary logistic regression, internally validated by bootstrapping and then externally validated.

**Results:**

Of 626 patients, 481 were included; 355 patients formed the model development group and 126 formed the external validation group. Strangulation occurred in 58 (16%) patients and non-operative treatment failed in 93 (31%) patients in development cohort. The following six variables were included in the risk model for strangulation and non-operative treatment failure: neutrophil–leucocyte ratio, number of previous SBOs, abdominal guarding, mesenteric changes and free abdominal fluid, closed loop sign, and faeces sign on CT. In the development cohort, the optimism corrected area under the receiver operator characteristics curve for the strangulation model was 0.860 (95% c.i. 0.808–0.917), and 0.751 (95% c.i. 0.694–0.816) for the non-operative treatment failure model respectively. At external validation, the models retained their discrimination and demonstrated stable calibration.

**Conclusion:**

A clinically relevant prediction model to predict strangulation and non-operative treatment failure in adhesive small bowel obstruction has been developed.

## Teaser

Small bowel obstruction is a leading cause of emergency surgery admission. Detecting patients at high risk of strangulation or failure of non-operative treatment is difficult. In this multicentre prospective observational study, we created prediction models for detection of strangulation and non-operative treatment failure in adhesive small bowel obstruction.

## Introduction

Small bowel obstruction (SBO) is a leading cause of emergency surgical admission^1–4^. Up to 60% of SBOs are due to peritoneal post-operative adhesions^5^. According to current Bologna guidelines, in the absence of concern about intestinal ischaemia (strangulation), adhesive SBO can be treated non-operatively with intravenous fluid administration, nasogastric (NG) tube insertion and oral water-soluble contrast (WSC) prescription^2^. If strangulation is suspected, immediate surgery is necessary^2^. However, detecting patients at high risk of strangulation or failure of non-operative treatment is difficult^6,7^. A recent national study (NASBO) from the UK concluded that timing of surgery could be one of the key modifiable factors to improve outcomes^3^. Several prediction models combining CT-scan findings, laboratory results, or clinical features to detect either strangulation or non-operative management failure have been generated^8–13^. However, they have usually had methodological issues that include small sample size, retrospective study design, and lack of calibration or external validation. At present, no prediction model appears to be widely used.

The overall mortality rate after laparotomy for adhesive SBO exceeds 7% and delay to emergency laparotomy more than 72 h after admission is associated with a higher 30-day postoperative mortality rate^4^ and longer hospital stay^14^. When non-operative management fails, the costs increase over seven-fold^15^. Well-performing prediction models that predict strangulation and non-operative treatment failure could help facilitate an earlier decision to operate and ultimately lead to better short- and long-term outcomes.

In this multicentre prospective observational study, two externally validated prediction models were developed: one for strangulation and the other for failure of non-operative management in adhesive SBO.

## Methods

The study was an observational prospective multicentre trial conducted in three hospitals in southern Finland: two university hospitals (Meilahti and Jorvi Hospitals, both part of Helsinki University Hospital) and one community hospital (Hyvinkää Hospital). The prediction models were developed using Meilahti hospital patient data and externally validated using Jorvi and Hyvinkää hospitals’ patient data. The study recruitment period was from 2 June 2014 to 31 March 2023 in Meilahti hospital and from 28 January 2018 to 31 March 2023 in Jorvi and Hyvinkää hospitals. The TRIPOD statement^16^ was applied for reporting this study and the checklist was completed (*Supplemental material*). Guidance regarding the development and validation of predictions models was also used^17–19^. The study has been registered in ClinicalTrials.gov (NCT03461744).

### Participants

Patients presenting to the surgical emergency department with CT-confirmed adhesive SBO with clinically relevant blood samples taken were included in the study. Patients under 18 years of age, pregnant patients, patients who had undergone abdominal surgery within 30 days, patients with inflammatory bowel disease, and patients with SBO caused by intraluminal obstruction, abdominal wall hernia, or peritoneal carcinomatosis were excluded. After inclusion to the trial no other trial interventions were performed and the patients were treated according to normal hospital protocols: if strangulation was suspected by the treating surgeon, emergency surgery was initiated. Otherwise, non-operative treatment was initiated. The non-operative treatment plan included intravenous hydration and NG-tube insertion, usually followed by WSC challenge after decompression. Any signs of strangulation during non-operative treatment were an indication for surgery. Urgent surgery was initiated if the SBO did not resolve, usually after WSC challenge. No informed consent was needed as this was an observational trial with no impact on patient care. The study protocol was approved by the Ethics Committee of Helsinki University Hospital on 26 March 2014, and by the Institutional Review Board of the Hospital District of Helsinki and Uusimaa. Patients were followed up from the medical records for 30 days from admission.

### Outcome

Primary outcomes for this study were strangulation defined by operative finding of any intestinal ischaemia (bowel necrosis or reversible ischaemia) and failure of non-operative treatment (need for surgery after initiation of non-operative management) within 30 days from admission.

### Predictors

The treating (on-call) physicians collected information about the patient history and symptoms, such as pain quality and severity, on a prospective case report form.

The following laboratory measurements were collected routinely from included patients: haemoglobin, leucocytes (white blood cell count), neutrophils, alanine aminotransferase, bilirubin, venous pH, venous base excess, lactate, creatinine kinase, fibrine D-dimer (FiDD), and sodium (Na).

The basic demographics of the patients including other diseases and medical conditions determined by the Elixhauser Co-morbidity index^20^ and previous abdominal operations were collected from medical records.

The CT scan images were re-analysed by two expert gastrointestinal radiologists (E.L. and H.P., radiology case report form presented in *Supplemental material*). The radiologists were not informed about the surgical findings.

In patients initiated on a non-operative treatment plan, the NG tube output in first 12 h was analysed using the nearest 12-hour mark value. Operative findings were analysed from patient records.

### Sample size

For the strangulation prediction model, full cohort was used for analyses. For the non-operative treatment failure prediction model, patients who underwent emergency surgery as initial treatment plan were excluded from analyses. It was estimated that at least 10 events were required for each predictor parameter, and the aim was to achieve 50 patients with strangulation in the development group. This dictated the eventual final sample size, which was not determined at the beginning of the study, as it depended on the strangulation rate in the final cohort.

### Missing data

Missing data were assumed to be missing at random and were imputed for the prediction model development using multiple imputation. Mice-package in R was used for imputation. Ten different imputed data sets were generated, and results reported are based on pooled analyses. The descriptive statistics were analysed before multiple imputation.

### Statistical analysis

Continuous variables are reported as means and standard deviations for normally distributed data and medians and interquartile ranges for data that are not normally distributed. Differences in variable distributions between cohorts, patients with or without strangulation, and patients with non-operative treatment failure or success were assessed using binary logistic regression, *t*-test or Mann–Whitney U-test for continuous variables and χ^2^ (2 × 2 tables with Yates’ continuity correction) or Fisher’s exact test for categorical variables.

Two-sided *P* < 0.050 was considered statistically significant. Data analysis was performed with SPSS^®^ version 28.0 for Macintosh (IBM, Armonk, NY, USA) and R (R Core Team, Vienna, Austria). The rms package in R was used (R package version 6.2-0).

#### Model development

Statistics-based variable selection (such as stepwise methods) leads easily to overfit where data set-specific noise is modelled in addition to the actual prediction problem, which potentially inflates the importance of certain predictor effects. To avoid this, variable selection was based on the combination of univariable analysis, previous literature, expert consensus, and clinical usefulness.

Continuous variables were not categorized. Possible non-linear associations were analysed with restricted cubic splines. The model was developed using binary logistic regression. Area under the receiver operator characteristics curve (AUROC) was used to assess model discrimination.

#### Model validation

Internal validation was performed by bootstrapping with 1000 resamples where apparent performance of the bootstrap samples and performance in the original data set (test performance) was calculated. Optimism was calculated as an average of apparent performance subtracted by test performance. Optimism corrected AUROC, Nagelkerke *R*^2^, calibration intercept, and calibration slope are calculated as apparent performance subtracted by optimism. Calibration intercept is a measure of the difference between average predicted probability and average observed probability and has a target value of 0 (no difference between the average values). Calibration slope evaluates the spread of estimated probabilities, that is how extreme the predictions are, and has a target value of 1 (<1 indicates too extreme predictions often resulting from overfit, >1 indicates too modest predictions)^21,22^. Optimism-adjusted calibration slope was used as a uniform shrinkage factor and shrunken regression coefficients and refitted intercept are used in external validation.

External validation was performed by analysing model discrimination and calibration in the combined Jorvi and Hyvinkää cohort. Calibration plots were drawn, and the slope and intercept were used to assess the calibration.

## Results

### Participants

**Fig. 1:**
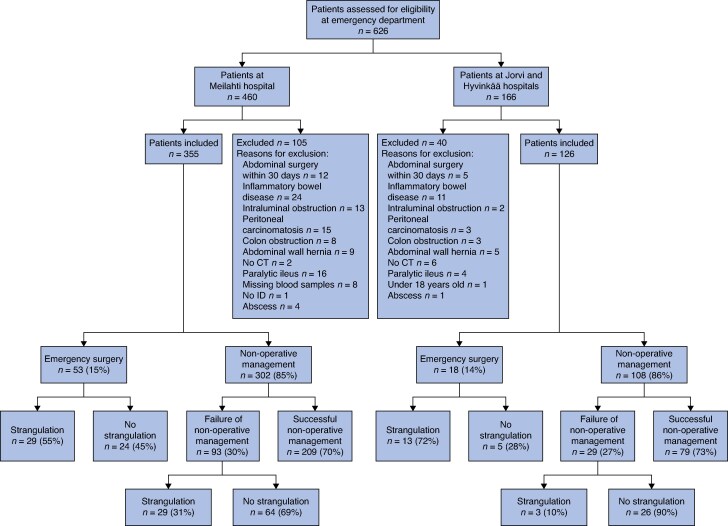
The study population

During the study period, a total of 626 patients were assessed for eligibility and 145 were excluded, leaving 481 patients in the analyses; 355 patients were identified in Meilahti hospital and formed the model development group; 126 patients were identified in Jorvi and Hyvinkää hospitals and formed the external validation group. *Figure 1* summarizes the study flow.

*Table 1* summarizes basic demographics and surgical details of patients in development and validation cohorts. A history of pelvic surgery was more common in the development group. Strangulation was more common after non-operative treatment trial in the development cohort. WSC challenge was initiated more often in the development cohort.

**Table 1:** Basic demographics and surgical details of patients in development and validation cohorts

	Development cohort *n* = 355	Validation cohort *n* = 126	*P*	Missing
Age, median(i.q.r.), years	70(58–79)	71(60–80)	0.434†	0
**Sex**				
Female	188 (53.0)	57 (45.2)	0.166*	0
BMI, median (i.q.r.), kg/m^2^	24.3(22.1–27.8)	25.0(22.3–28.3)	0.231†	51(10.6)
Co-morbidities, median (i.q.r.), Elixhauser score	2(1–3)	2(0–3)	0.780†	1(0.2)
Previous abdominal operation	303 (85.6)	101 (80.8)	0.261*	2 (0.4)
History of pelvic surgery	211 (59.8)	61 (48.8)	**0**.043*	3 (0.6)
Previous SBO	117 (32.3)	48 (38.7)	0.322*	5 (1.0)
Number of previous SBOs, median(i.q.r., range)	0(0–1; 0–20)	0(0–1; 0–35)	0.316†	5(1.0)
Immediate surgery	53 (14.9)	18 (14.3)	0.977*	0
Strangulation	58 (16.3)	16 (12.7)	0.407*	0
Strangulation finding when immediate surgery	29 (54.7)	13 (72.2)	0.304*	0
Non-operative treatment failure	93 (30.8)	29 (26.9)	0.518*	0
Strangulation finding after non-operative treatment failure	29 (31.2)	3 (10.3)	**0**.047*	0
Water-soluble contrast challenge	249 (70)	69 (55)	**0**.003*	2 (0.4)
Surgery during admission	137 (38.6)	45 (35.7)	0.642*	0
Surgery in 30 days from admission	151 (42.9)	47 (37.9)	0.387*	5 (1.0)
Bowel resection	43 (29.1)	18 (39.1)	0.270*	0
Bowel resection length, median(i.q.r.), cm	20(10–53)	50(10–100)	0.118†	1(0.2)
Readmission in 30 days	46 (13.8)	14 (11.5)	0.627*	25 (5.2)
Mortality rate in 30 days	18 (5.1)	4 (3.2)	0.527*	1 (0.2)

Categorical variables are marked as *n* (%) and analysed with chi-square test. *2 × 2 tables with Yates’ continuity correction. Continuous variables are analysed with Mann–Whitney U-test †and presented either median(i.q.r.) or mean(s.d.) respectively. Missing values are reported as *n* (%). *P*-values <0.05 are bolded.

### Model development and validation

#### Prediction model for strangulation: the strangulation risk score

In the development cohort, 58 (16%) of patients had strangulation. Of those patients, strangulation was diagnosed at emergency surgery immediately after presentation in 29 (50%) patients and at surgery after non-operative treatment failure in 29 (50%) patients. The remaining 297 patients had either successful non-operative treatment (*n* = 209, 70%) or surgery without bowel ischaemia (*n* = 88, 30%). Patient history, clinical examination findings, laboratory results, and CT imaging findings were analysed and are shown in *Table S1*.

Some of the variables, although associated statistically with strangulation, were excluded from the prediction model development. Variables that are not routinely readily available, highly subjective, or challenging to assess or accrue in emergency room setting, such as FiDD or bowel auscultation, were excluded. In addition, variables with very few events, such as pneumatosis or peritoneal gas, were excluded. The final variable selection was based on univariable analysis, previous literature, and clinical usefulness. *Table S2* illustrates the variable selection process. As the number of strangulations was 58, six predictors could be added to the model. The following predictors were deemed most relevant: neutrophil to leucocyte ratio (NL ratio), number of previous SBOs (range 0–5), abdominal guarding (peritonism or defence *versus* diffuse tenderness or no tenderness), closed loop sign on CT, faeces sign in CT, and three class combination predictor utilizing mesenterial fat stranding/oedema and free abdominal fluid in CT. NL ratio was modelled as a linear term as using restricted cubic splines did not differ significantly (*Fig. S1*).

The final strangulation prediction model had an apparent AUROC of 0.876 (95% c.i. 0.828 to 0.925) and Nagelkerke *R*^2^ of 0.437 in the development cohort, and an optimism-corrected AUROC of 0.860 (95% c.i. 0.808 to 0.917) after internal validation, and Nagelkerke *R*^2^ of 0.389. The optimism-adjusted calibration slope was 0.89, indicating tendency for slight overfit. This was used as a shrinkage factor to re-estimate regression model coefficient (shrinkage factor × original coefficient). *Table 2* shows the original and shrunken model coefficients and performance measurements.

**Table 2:** Strangulation model (STRISK) specifics and results of internal and external validation

Predictor	Range/unit/coding	Coefficients	Standard error	Shrunken coefficient (shrinkage factor = 0.89)	OR (95% c.i.) (final model)
Previous SBO events	0–5	−0.4608	0.32	−0.4101	0.66 (0.38–1.16)
Abdominal guarding	0 = none/diffuse tenderness, 1 = guarding/peritonism	0.8408	0.38	0.7482	2.11 (1.08–4.12)
NL ratio	0–100%	0.0363	0.018	0.0322	1.03 (1.00–1.06) per 1 unit of increase
Faeces sign	1 = positive, 0 = negative	−1.1513	0.48	−1.0246	0.36 (0.15–0.83)
Mesenterial oedema/abdominal fluid	2 = free abdominal fluid, 1 = mesenterial oedema (no free fluid), 0 = negative	1 = 1.3282 2 = 2.1752	1 = 1.13 2 = 1.09	1 = 1.1821 2 = 1.9359	1 = 3.26 (1.23–8.64) 2 = 6.93 (2.71–17.74)
Closed loop sign	1 = positive, 0 = negative	1.9522	0.36	1.7374	5.68 (3.02–10.69)
Intercept		−6.8966	1.84	−6.2487	
**Performance**		**Apparent**		**Optimism-corrected**	**External**
Apparent AUC		0.876 (0.828–0.925)		0.860 (0.808–0.917)	0.907 (0.822–0.993)
Nagelkerke *R*^2^		0.438		0.389	0.536
Calibration slope		1.0		0.89	1.29
Calibration intercept		0		0.0	−0.15

**Fig. 2:**
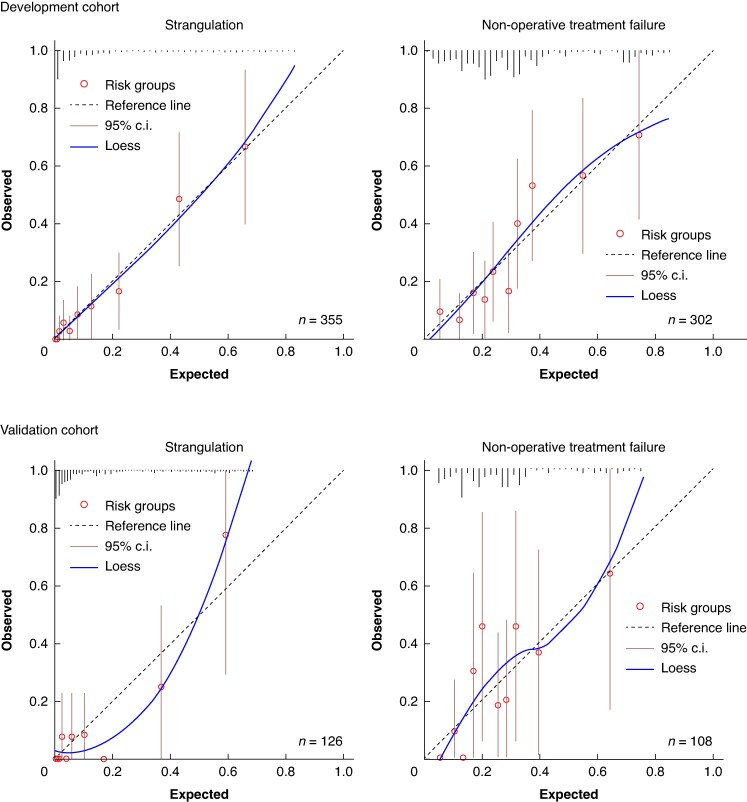
Apparent and external calibration for STRISK and NOFA models

The internally validated and optimism-corrected model performance was then assessed in an external cohort. The model retained excellent discrimination with an AUROC of 0.907 (95% c.i. 0.822 to 0.993). The calibration slope and intercept demonstrated adequate calibration (1.29, −0.15 respectively), with slight average risk overestimation and slight risk underestimation for high-risk patients in this validation cohort (*Fig. 2*).

Predicted probability on *x*-axis, actual probability on *y*-axis, perfectly calibrated prediction model would have a diagonal calibration curve (reference line). Histograms represent the distribution of predicted risk.

#### Prediction model for non-operative treatment failure: the non-operative treatment failure score

In the development cohort, non-operative treatment failed in 93 (31%) patients (of 302 patients initially treated non-operatively). The comparison between patients with non-operative treatment failure and success is shown in *Table S3*.

For the development of the non-operative treatment failure (NOFA) model, the same variables were used as for the strangulation risk (STRISK) model as they were deemed clinically relevant and reliable to measure (*Tables S2*, *S4*) and the combined model use would be easier with the same variables. STRISK-model regression coefficients were not viable for predicting non-operative treatment failure as the patient population is selected and has a lower baseline risk for strangulation and the model should predict also non-resolving SBO without strangulation. The STRISK model demonstrated sufficient discrimination, AUROC = 0.767 (95% c.i. 0.670 to 0.864), but inadequate calibration (intercept = 1.821, slope = 0.746) for predicting non-operative treatment failure (*Fig. S2*). Thus, a model with the same six variables was fitted in a cohort consisting of patients undergoing the initial non-operative treatment plan in Meilahti Hospital (*n* = 302) and validated in patients begun on non-operative treatment plan in Jorvi and Hyvinkää Hospitals (*n* = 108).

The NOFA model demonstrated an apparent AUROC of 0.773 (95% c.i. 0.715 to 0.831) and Nagelkerke *R*^2^ of 0.248. The optimism-corrected values were 0.751 (95% c.i. 0.694 to 0.816) and 0.205 respectively. Optimism-adjusted calibration slope (0.88) was again used as a shrinkage factor. In the validation cohort, the model retained its discrimination with an AUROC of 0.751 (95% c.i. 0.653 to 0.850) and demonstrated stable calibration with slope and intercept of 1.10 and 0.07 respectively (*Fig. 2*). *Table 3* shows the original and shrunken model coefficients and performance measurements.

**Table 3:** Failure of non-operative treatment model (NOFA) specifics, internal and external validation

Predictor	Range/unit/coding	Coefficients	Standard error	Shrunken coefficient (shrinkage factor = 0.88)	OR (95% c.i.) (final model)
Previous SBO events	0–5	−0.3835	0.14	−0.3385	0.71 (0.56–0.91)
Abdominal guarding	0 = none/diffuse tenderness, 1 = guarding/peritonism	0.4208	0.34	0.3714	1.45 (0.81–2.61)
NL ratio	0–100%	0.0063	0.012	0.0055	1.01 (0.99–1.03) per 1 unit of increase
Faeces sign	1 = positive, 0 = negative	−0.4770	0.31	−0.4210	0.67 (0.38–1.12)
Mesenterial oedema/abdominal fluid	2 = free abdominal fluid, 1 = mesenterial oedema (no free fluid), 0 = negative	1 = 0.9132 2 = 0.6374	1 = 0.45 2 = 0.41	1 = 0.8060 2 = 0.5626	1 = 2.24 (1.52–3.30)) 2 = 1.76 (1.23–2.50)
Closed loop sign	1 = positive, 0 = negative	1.6344	0.35	1.4426	4.23 (2.31–7.74)
Intercept		−1.8809	0.96	−1.7437	
**Performance**		**Apparent**		**Optimism-corrected**	**External**
Apparent AUC		0.773 (0.715–0.831)		0.751 (0.694–0.816)	0.751 (0.653–0.850)
Nagelkerke *R*^2^		0.248		0.200	0.201
Calibration slope		1.0		0.88	1.10
Calibration intercept		0		0.03	0.07

Final model equations are presented in the supplemental information.

Both models can be found as web-based calculator from www.tinyurl.com/strisk.

#### Classification

**Fig. 3:**
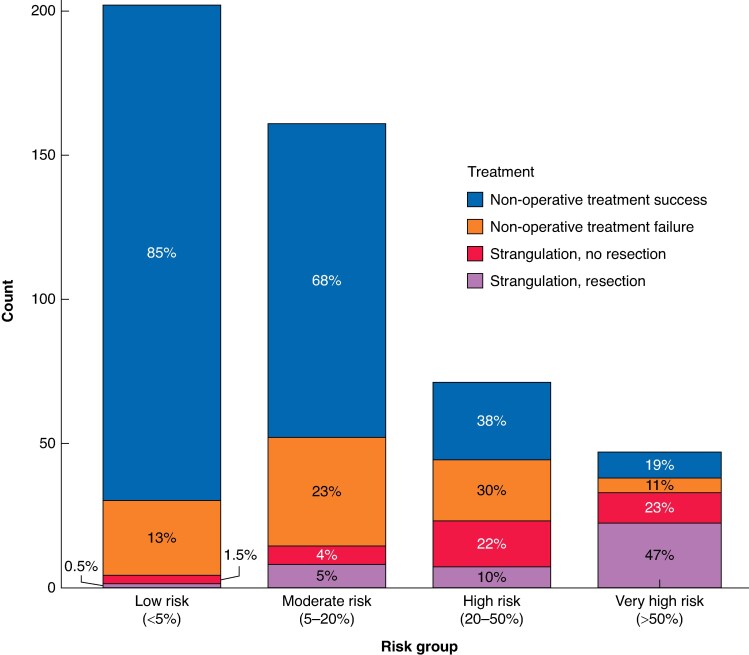
The illustration of outcome status of patients in a cohort combining development and validation cohort patients, risk stratification by STRISK model predictions

To demonstrate the models’ ability to classify patients, receiver operating characteristic (ROC) curves were drawn for both models in a cohort combining development and validation cohort patients to maximize sample size. Cut-off points for higher specificity or higher sensitivity were visually selected from the curve and the metrics are presented in *Fig. S3*. These cut-off points simulate possible clinical decision-making thresholds. *Figure 3* illustrates the outcome status of patients stratified by strangulation-model predictions in a cohort combining development and validation cohort patients. The proportion of patients who needed surgery as well as intraoperative findings of strangulation and need for bowel resection increased in four illustrated risk groups from low risk to very high risk.

Non-operative treatment failure includes patients whose non-operative treatment trial failed and needed surgery but did not have strangulation. Strangulation is divided to strangulation finding with or without bowel resection.

## Discussion

Early recognition of strangulation or possible need for surgery in patients with SBO is essential to facilitate safe surgical or non-operative management. Several models to predict either need for surgery, strangulation, or failure of non-operative treatment have been developed. However, these models have lacked important features such as internal validation and calibration of the models. Thus, their repeatability is low. In this prospective multicentre study, two prediction models were developed and externally validated (STRISK and NOFA) to help select the most appropriate treatment method for patients with adhesive SBO.

Predictors utilized in both models are number of previous SBO events, abdominal guarding, NL ratio, small bowel faeces sign, mesenteric oedema and free abdominal fluid, and closed loop sign. The first episode of SBO is a known risk factor for strangulation^23^ with the probability of needing surgery decreasing with each new SBO episode^24^. In addition, previous episode of adhesive SBO has been found to be protective against the failure of non-operative management^11^. In this study, an almost linear negative association between the number of previous SBO events and both strangulation and non-operative treatment failure was observed. Abdominal guarding is a sign of peritoneal irritation and traditionally has been thought to be associated with strangulation. Guarding was associated with need for bowel resection in a recent study^23^ and risk for surgery in a recent meta-analysis^25^. The NL ratio has been widely studied in different conditions and is found to be associated with strangulation in SBO^26^ and also strangulation of inguinal hernia^27^. In this study the NL ratio was used as it is usually available at initial presentation. Of note, NL ratio had slightly better predictive value over leucocyte count alone, which is currently recommended in the Bologna guidelines^2^. Small bowel faeces sign is well reported in the SBO literature and was associated with lower probability of strangulation and higher likelihood of successful non-operative management in our study. The mechanism of this phenomenon is hypothesized to be due the small bowel’s ability to resorb fluids in less severe obstruction, leading to faeces-like small bowel contents^9^. The lack of a small bowel faeces sign has previously been found to be predictive sign of strangulation^9,10^, failure of non-operative management^7^, and need for surgery^25,28^. Mesenteric oedema and fluid have been confirmed as predictors for strangulation^29,30^ and absence of mesenteric fluid has been a reliable finding to rule out strangulation^31^. Free peritoneal fluid has also been demonstrated to predict strangulation^23,25,32^ and also a sign of the need for operative treatment^9,13,25,28,33^. Mesenteric changes (mesenteric stranding or oedema) and abdominal fluid can be thought as a continuum and express the severity of adhesive SBO. In this study a three step variable combining these CT-findings was constructed. A closed loop sign was the strongest predictor for both strangulation and failure of non-operative management as previously recognized in multiple studies^9,11,25,34^. In this situation, the formation of a small bowel segment without proximal and distal outlets for decompression may lead to rapid compromise of the bowel wall blood supply. However, there have been studies demonstrating closed loop SBO can resolve without surgery, and distance between the transition zones should also be assessed^35,36^.

**Fig. 4:**
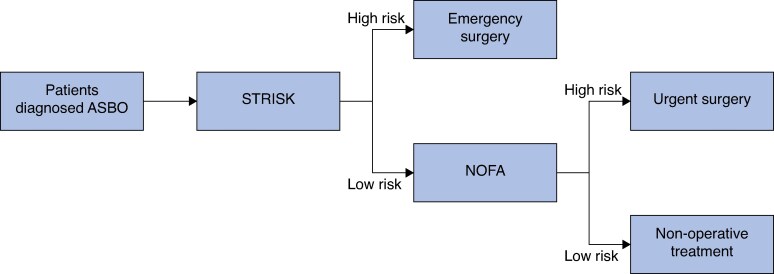
The flowchart of the potential use of the models and treatment strategy

STRISK, strangulation risk score; NOFA, non-operative treatment failure score.

Timely surgical intervention is the cornerstone of adhesive SBO treatment. A flowchart for the potential use of the models is presented in *Fig. 4*. Use of the models can lead to an increased number of surgical operations. In fact, the strangulation score seemed to slightly overestimate the average risk for strangulation in the validation cohort in the moderate risk range, which can be explained partly by the model’s innate uncertainty and partly by slightly lower strangulation incidence in the validation group. Nevertheless, the overall calibration remained good. In addition, recent studies favour operative treatment options, at least for patients with their first episode of adhesive SBO to reduce the risk of recurrence^24,37,38^ and costs^39^.

The strengths of this study were its prospective design and external validation in two hospitals, differing both in their academic status and treatment volume. In addition, prediction model variables were selected based on combination of univariable analysis, expert consensus, and previous literature.

This study had some limitations. Although this was a prospective study, there were missing values, especially in laboratory results. However, the percentage of missing values for variables used in prediction model development was low and could be reliably imputed. The distance of the closed loop’s transition zones was not analysed. The external validation cohort was small with only 16 cases of strangulation and 29 cases of failed non-operative treatment, rendering the reliability of external validation suboptimal.

The presented prediction models may help in clinical decision-making to determine which adhesive SBO patients need immediate or urgent surgery. The optimization of selecting patients for urgent surgical treatment might decrease the mortality and morbidity rates associated with adhesive SBO and shorten the length of hospital stay. To investigate the actual impact in clinical practice, prospective implementation studies are needed.

## Supplementary Material

znaf025_Supplementary_Data

## Data Availability

The study permissions and Finnish law do not allow individual patient data sharing.
