# Supplementary material for: Development and external validation of prediction risk scores (STRISK and NOFA) to predict immediate surgical need in adhesive small bowel obstruction: an observational prospective multicentre study
Source: Br J Surg. 2025 Mar 19;112(3):znaf025. doi: 10.1093/bjs/znaf025 (PMC11921420; doi:10.1093/bjs/znaf025)
Supplement: znaf025_Supplementary_Data [file znaf025_supplementary_data.zip › SBO_Supplement_Radiology_case_report_form.docx]

Radiology case report form

Patient information

Name: __________________________

Social security number: _____________

CT-scan

- Technical details:
  - Scan time___/___/20___ time: ____: ____
  - Scanner model: ______________________
    - Siemens Definition AS+
    - GE Medical systems VCT
    - GE Medical Systems Light Speed VCT
    - Siemens Somatom Definiton Flash
    - Siemens Somatom Edge
    - Siemens Somatom AS
    - Toshiba Aquilon Prime
    - GE Medical Systems Revolution Evo
    - GE Medical Systems Light Speed Ultra
    - GE Medical Systems Discovery CT 750 HD
    - GE Medical Systems Revolution HD
  - I.V. contrast ☐ yes ☐ no
  - P.O. contrast ☐ Water ☐ Gastrografin ☐ No
- Findings:
  - Small bowel gas-fluid levels ☐ yes ☐ no
  - Transition site ☐ yes ☐ no

Number of transition sites: ____

Most proximal transition site ☐ Jejunum ☐ Ileum ☐ Not detectable

- - Small bowel maximum diameter ____mm
  - Closed loop sign ☐ yes ☐ no
  - Normal IV-contrast enhancement of the small bowel wall ☐ yes ☐ no
    - No contrast enhancement ☐ yes ☐ no
    - Poor contrast enhancement ☐ yes ☐ no
    - Intense contrast enhancement ☐ yes ☐ no
  - Small bowel mesentery ☐ Normal ☐ Fat stranding ☐ Edema
  - Whirl sign ☐ yes ☐ no
  - Pneumatosis of bowel wall ☐ yes ☐ no
  - Gas in portomestenteric veins ☐ yes ☐ no
  - Small bowel feces sign ☐ yes ☐ no
  - Peritoneal gas

☐ yes, big bubbles (over 1x1cm or 2cm in any direction)

☐ yes, small bubbles (under 1x1cm and 2cm in any direction)

☐ no

- - Peritoneal fluid ☐ yes ☐ no
  - Fluid density _________HU

Water soluble contrast challenge

- - Performed ☐ yes ☐ no
  - Passage time___/___/20___ time: ____: ____ (2h scan)
  - 2h scan
    - Contrast in ventricle ☐ yes ☐ no
    - Contrast in small bowel ☐ yes ☐ no
    - Contrast in colon ☐ yes ☐ no
  - 8h scan
    - Contrast in ventricle ☐ yes ☐ no
    - Contrast in small bowel ☐ yes ☐ no
    - Contrast in colon ☐ yes ☐ no
