# Supplementary material for: Development and external validation of prediction risk scores (STRISK and NOFA) to predict immediate surgical need in adhesive small bowel obstruction: an observational prospective multicentre study
Source: Br J Surg. 2025 Mar 19;112(3):znaf025. doi: 10.1093/bjs/znaf025 (PMC11921420; doi:10.1093/bjs/znaf025)
Supplement: znaf025_Supplementary_Data [file znaf025_supplementary_data.zip › Supplementary_material.docx]

**Development and external validation of prediction risk scores (STRISK and NOFA) to predict immediate surgical need in adhesive small bowel obstruction: an observational prospective multicenter study**

Panu Räty, MD^1^; Akseli Bonsdorff, MD, PhD^1^; Helka Parviainen, MD, PhD^2^; Eila Lantto, MD, PhD^2^; Thomas Hackenberg, MD^3^; Hanna Lampela, MD, PhD^1^, Taina Nykänen MD, PhD^3^; Ilana Lyytinen, MD; Panu Mentula, MD, PhD^1^; Ville Sallinen, MD, PhD^1,5^

^1^ Gastroenterological Surgery, Helsinki University Hospital and University of Helsinki, Helsinki, Finland

^2^ HUS Medical Imaging Center, Radiology, Helsinki University Hospital and University of Helsinki, Finland

^3^ Department of Surgery, Hyvinkää Hospital, Hyvinkää, Finland

^4^ Department of Surgery, Kanta-Häme Central Hospital, Finland

^5^ Department of Transplantation and Liver Surgery, Helsinki University Hospital and University of Helsinki, Helsinki, Finland

**Corresponding author** Ville Sallinen, MD, PhD, Gastroenterological Surgery, University of Helsinki, and Helsinki University Hospital, Haartmaninkatu 4, 00029 Helsinki, Finland, [ville.sallinen@helsinki.fi](mailto:ville.sallinen@helsinki.fi), ORCiD id: 0000-0001-5394-4169

**Supplementary Materials - Index**

| **Supplementary Results** |  |
| --- | --- |
| The Final Model Equations | *page 2* |
| **Supplementary Figures and Tables** |  |
| Table S1 | *page 3-5* |
| Table S2 | *page 6-7* |
| Table S3 | *page 8-10* |
| Table S4 | *page 11-12* |
| Figure S1 | *page 13* |
| Figure S2 | *page 14* |
| Figure S3 | *page 15* |
| **References** | *page 16-17* |
|  |  |

**The final model equations**

$P(STRISK model)=(\exp(- 6.2487-0.4101[number of previous SBO events)+0.7482[defance or peritonism]+0.0322[NL-ratio]-1.0246[feces sign]+(1.1821\left[ mesenterial edema \right] OR 1.9359\left[ free abdominal fluid \right] )+1.7374[closed loop])/(1+\exp\left( - 6.2487-0.4101\left[ number of previous SBO events \right)+0.7482\left[ defance or peritonism \right]+0.0322\left[ NL-ratio \right]-1.0246\left[ feces sign \right]+\left( 1.1821\left[ mesenterial edema \right] OR 1.9359\left[ free abdominal fluid \right] \right)+1.7374\left[ closed loop \right] \right)))$

$P(NOFA model)=(\exp(- 1.7437-0.3385[number of previous SBO events)+0.3714[defance or peritonism]+0.0055[NL-ratio]-0.4210[feces sign]+(0.8060\left[ mesenterial edema \right] OR 0.5626\left[ free abdominal fluid \right] )+1.4426[closed loop])/(1+(\exp(- 1.7437-0.3385\left[ number of previous SBO events \right)+0.3714\left[ defance or peritonism \right]+0.0055\left[ NL-ratio \right]-0.4210\left[ feces sign \right]+\left( 0.8060\left[ mesenterial edema \right] OR 0.5626\left[ free abdominal fluid \right] \right)+1.4426\left[ closed loop \right])))$

**Table S1** Comparison of patients with strangulation and no strangulation in the development cohort. Categorical variables are marked as n (%) and analyzed either with binary logistic regression, Chi-Square test (a) (2x2 tables with Yates' continuity correction) or Fisher's exact test (b). Continuous variables are analyzed with binary logistic regression and presented either median (IQR) or mean (SD), respectively. Missing values are reported as n (%).

BMI – body mass index, SBO – small bowel obstruction, MAP – mean arterial pressure, Hb – hemoglobin, WBC – white blood cell count, CRP – C-reactive protein, BE – base excess, FiDD – fibrin D-dimer, CK – creatine kinase, ALT – alanine aminotransferase, CRP – C-reactive protein.

|  |  | | **Strangulation (n=58)** | **No strangulation (n=297)** | **OR (95% CI)** | **p** | **Missing** |
| --- | --- | --- | --- | --- | --- | --- | --- |
| **Basic demographics** |  |  | | | | | |
| Age, median (IQR), years |  | | 76 (62-85) | 69 (57-77) | 1.02 (1.00-1.04) | 0.081 | 0 |
| Women |  | | 42 (72.4) | 146 (49.2) | **2.72 (1.46-5.04)** | **0.002** | 0 |
| BMI, median (IQR), kg/m^2^ |  | | 22.9 (20.7-25.0) | 24.3 (22.0-28.4) | **0.90 (0.84-0.96)** | **0.003** | 34 (9.6) |
| Comorbidities (Elixhauser score, median (IQR)) |  | | 1 (1-2) | 2 (0-3) | 0.91 (0.74-1.11) | 0.349 | 1 (0.3) |
| Previous abdominal operation |  | | 46 (78.9) | 258 (86.9) | 0.57 (0.28-1.17) | 0.122 | 1 (0.3) |
| Number of previous abdominal operations (IQR) |  | | 1 (1-2) | 2 (1-3) | 0.84 (0.69-1.02) | 0.080 | 1 (0.3) |
| Previous open abdominal operation |  | | 41 (71.9) | 239 (80.7) | 0.61 (0.32-1.17) | 0.135 | 2 (0.6) |
| Previous pelvic surgery |  | | 35 (61.4) | 176 (59.5) | 1.09 (0.61-1.94) | 0.784 | 2 (0.6) |
| Abdominal radiotherapy |  | | 2 (3.4) | 28 (9.4) | 0.34 (0.08-1.48) | 0.152 | 0 |
| Previous SBO |  | | 6 (10.3) | 111 (37.8) | **0.19 (0.08-0.46)** | **<0.001** | 3 (0.8) |
| Number of previous SBOs, median (IQR; range) |  | | 0 (0; 0-2) | 0 (0-1; 0-20) | **0.34 (0.18-0.66)** | **0.001** | 3 (0.8) |
| **History and physical** |  |  | | | | | |
| Pain severity, median (IQR), range 0-10 |  | | 6 (3-8) | 4 (1-7) | **1.12 (1.02-1.23)** | **0.013** | 27 (7.6) |
| Colic pain |  | | 30 (53.6) | 168 (57.5) | 0.85 (0.48-1.51) | 0.584 | 7 (2.0) |
| Constant pain |  | | 32 (57.1) | 138 (47.3) | 1.49 (0.84-2.65) | 0.177 | 7 (2.0) |
| Vomiting |  | | 48 (82.8) | 224 (76.2) | 1.50 (0.72-3.12) | 0.278 | 3 (0.8) |
| Vomiting gastric type content |  | | 43 (81.1) | 169 (70.7) | 1.78 (0.85-3.74) | 0.128 |  |
| Vomiting fecal content |  | | 5 (33.3) | 55 (44.0) | 0.64 (0.21-1.97) | 0.433 |  |
| Duration from bowel movement, median (IQR), hours |  | | 31 (17-59) | 28 (13-56) | 1.00 (1.00-1.01) | 0.361 | 24 (6.8) |
| Abdominal guarding (defense or peritonism) |  | | 24 (41.4) | 57 (19.2) | **2.97 (1.64-5.40)** | **<0.001** | 0 |
| Bowel sounds | Normal | | 15 (26.3) | 88 (29.7) | 0.84 (0.45-1.60) | 0.604 | 2 (0.6) |
|  | Silent | | 26 (45.6) | 104 (35.1) | 1.55 (0.87-2.75) | 0.135 | 2 (0.6) |
|  | Metallic | | 7 (12.3) | 60 (20.3) | 0.55 (0.24-1.28) | 0.164 | 2 (0.6) |
|  | Splashing | | 15 (26.3) | 85 (28.7) | 0.89 (0.47-1.68) | 0.713 | 2 (0.6) |
| MAP, mean (SD) |  | | 96 (13) | 99 (13) | 0.98 (0.96-1.00) | 0.108 | 0 |
| Pulse, mean (SD) |  | | 83 (18) | 82 (15) | 1.00 (0.99-1.02) | 0.503 | 1 (0.3) |
| Temperature, median (IQR) |  | | 37.0 (36.7-37.2) | 37.0 (36.6-37.3) | 1.18 (0.69-2.02) | 0.537 | 0 |
| **Laboratory** |  |  | | | | | |
| Hb, mean (SD), g/l |  | | 136 (14) | 140 (19) | 0.99 (0.97-1.00) | 0.078 | 1 (0.3) |
| WBC, median (IQR), E9/l |  | | 11 (9.1-15.1) | 10 (7.5-12.4) | **1.08 (1.01-1.15)** | **0.020** | 0 |
| Neutrophils, median (IQR), E9/l |  | | 9.4 (6.8-13.7) | 7.5 (5.3-10.1) | **1.11 (1.03-1.19)** | **0.004** | 31 (8.7) |
| Neutrophil-WBC ratio, median (IQR), % |  | | 84 (72.7-89.2) | 77 (68.2-83.5) | **1.06 (1.02-1.09)** | **0.001** | 31 (8.7) |
| Thrombocytes, median (IQR), E9/l |  | | 239 (200-274) | 236 (196-283) | 1.00 (0.99-1.00) | 0.271 | 0 |
| CRP, median (IQR), mg/l |  | | 11 (5-42) | 12 (4-36) | 1.00 (1.00-1.01) | 0.355 | 0 |
| Na, median (IQR), mmol/l |  | | 136 (134-139) | 138 (135-140) | 0.97 (0.91-1.02) | 0.234 | 0 |
| Lactate, median (IQR), mmol/l |  | | 1.4 (1.2-2.0) | 1.2 (1.0-1.7) | **1.51 (1.10-2.06)** | **0.010** | 38 (10.7) |
| pH, median (IQR) |  | | 7.38 (7.35-7.44) | 7.39 (7.35-7.42) | 1.00 (0.93-1.06)/0.01 units | 0.907 | 53 (14.9) |
| BE, median (IQR), mmol/l |  | | 2.7 (-.2-4.8) | 2.2 (-.3-4.3) | 1.03 (0.95-1.12) | 0.422 | 53 (14.9) |
| FiDD, median (IQR), mg/l |  | | 1.7 (1.0-2.9) | 1.0 (.5-1.9) | 1.06 (0.98-1.14 | 0.159 | 46 (13.0) |
| CK, median (IQR), U/l |  | | 83 (44-124) | 80 (47-125) | 1.00 (1.00-1.00) | 0.455 | 48 (13.5) |
| ALT, median (IQR), U/l |  | | 16 (12-24) | 21 (13-30) | 0.98 (0.96-1.00) | 0.103 | 12 (3.4) |
| Bilirubin, median (IQR), µmol/l |  | | 12 (10-21) | 13 (9-16) | 1.01 (0.98-1.03) | 0.682 | 14 (3.9) |
| **Radiology** |  |  | | | | | |
| Number of transition sites |  | | 2 (1-2) | 1 (1) | **2.71 (1.79-4.10)** | **<0.001** | 30 (8.5) |
| Most proximal transition site | No | | 0 (0) | 14 (5) |  | 0.409^a^ | 8 (3.5) |
|  | Jejunum | | 13 (23) | 65 (22) |  |  |  |
|  | Ileum | | 42 (73) | 202 (70) |  |  |  |
|  | Unclear | | 2 (4) | 9 (3) |  |  |  |
| Small bowel maximum diameter, median (IQR, range), mm |  | | 34 (30-42) | 41 (36-46) | **0.91 (0.87-0.95)** | **<0.001** | 0 |
| Closed loop sign |  | | 42 (72.4) | 48 (15.8) | **13.96 (7.26-26.9)** | **<0.001** | 0 |
| Poor bowel wall contrast enhancement |  | | 21 (43.8) | 13 (5.3) | **14.00 (6.30-31.10)** | **<0.001** | 60 (16.9) |
| No contrast enhancement in bowel wall |  | | 2 (4.2) | 1 (0.4) | 10.65 (0.95-119.92) | 0.055 | 61 (17.2) |
| Intense enhancement of contrast in bowel wall |  | | 2 (4.2) | 4 (1.6) | 2.64 (0.47-14.85) | 0.270 | 60 (16.9) |
| Whirl sign |  | | 10 (17.2) | 31 (10.5) | 1.77 (0.82-3.86) | 0.148 | 2 (0.6) |
| Mesenteric edema or fluid |  | | 53 (91.4) | 198 (66.9) | **5.25 (2.03-13.54)** | **<0.001** | 1 (0.3) |
| Feces sign |  | | 8 (13.8) | 117 (39.4) | **0.25 (0.11-0.54)** | **<0.001** | 0 |
| Peritoneal fluid |  | | 45 (77.6) | 146 (49.3) | **3.56 (1.84-6.87)** | **<0.001** | 1 (0.3) |
| Pneumatosis of bowel wall |  | | 1 (1.7) | 1 (0.3) | 5.16 (0.32-83.67) | 0.248 | 2 (0.6) |
| Peritoneal gas (small bubbles) |  | | 0 (0) | 3 (1.0) |  | 1.00^b^ | 2 (0.6) |

**Table S2** Variable selection to the strangulation risk (STRISK) prediction model. Marking with “x” if determined important by univariable analysis, literature (variable associated with strangulation or need for surgery in previous literature) or by clinical assessment. Marked “(x)” if potentially difficult to use clinically. Variables included to the model are bolded. Mesenteric edema or fluid and peritoneal fluid are included as combined variable.

SBO – small bowel obstruction, BMI – body mass index, WBC – white blood cell count, FiDD – fibrin D-dimer, ALT – alanine aminotransferase, PCT – procalcitonin.

| **Variable** | **Univariable analysis suggests** | **Literature suggests** | **Clinically useful** | **Argument against of use in prediction model** | **Literature reference** |
| --- | --- | --- | --- | --- | --- |
| Age | x | x | (x) | Multicollinearity | (1, 2) |
| Sex | x |  | (x) | Multicollinearity |  |
| Previous SBO | x | x | x |  | (2) |
| **Number of previous SBOs** | x | x | x |  | (3) |
| Vomiting |  | x |  | Very common in SBO patients | (4) |
| Obstipation |  | x |  |  | (2) |
| Pain duration |  | x | (x) |  | (1, 5) |
| BMI | x |  |  |  |  |
| Tachycardia |  | x | (x) | Multicollinearity | (6) |
| Pain severity | x |  | (x) | Subjective |  |
| **Abdominal guarding** | x | x | x |  | (2, 4, 5, 7, 8) |
| WBC | x | x | x |  | (1, 5, 6, 8) |
| Neutrophils | x |  | x |  |  |
| **Neutrophil-WBC -ratio** | x |  | x |  |  |
| Neutrophil-lymphocyte ratio |  | x |  |  | (9) |
| Lactate | x | x | (x) | May not be routinely in use | (4) |
| FiDD | x |  | (x) | May not be routinely in use |  |
| ALT | x |  | (x) | May not be routinely in use |  |
| CRP |  | x | x |  | (2, 5) |
| PCT |  | x |  | PCT not in routine use in Finland | (10) |
| Na |  | x | (x) | Affected by numerous medical conditions | (11) |
| Transition site |  | x |  | Very common in adhesive SBO | (2) |
| Number of transition sites | x |  | x |  |  |
| **Closed loop sign** | x | x | x |  | (4, 7, 11-13) |
| Small bowel maximum diameter | x |  | x |  |  |
| Bowel wall poor contrast loading | x | x | x | Usually leads to exploration | (1, 2, 5, 7, 8, 12, 14, 15) |
| **Mesenteric edema or fluid** | x | x | x |  | (1, 4, 6, 7, 12-16) |
| **Peritoneal fluid** | x | x | x |  | (2, 4-7, 13, 14, 16) |
| **Feces sign** | x | x | x |  | (4, 13, 16) |
| Peritoneal fluid density | x | x | x |  | (17) |
| Thickened bowel wall |  | x | x |  | (7, 11, 14) |
| Peritoneal air |  | x | (x) | Usually leads to exploration | (7) |
| Whirl sign |  | x | x |  | (14) |
| The degree of obstruction |  | x |  | Not useful with modern CT scan imaging | (16) |
| Pneumatosis |  | x | (x) | Usually leads to exploration | (4) |
| Portal venous gas |  | x | (x) | Usually leads to exploration | (4) |

**Table S3** Comparison of patients with nonoperative treatment failure and success in the development cohort. Categorical variables are marked as n (%) and analyzed either with binary logistic regression or Fisher's exact test (b). Continuous variables are analyzed with binary logistic regression and presented either median (IQR) or mean (SD), respectively. Missing values are reported as n (%).

BMI – body mass index, SBO – small bowel obstruction, MAP – mean arterial pressure, Hb – hemoglobin, WBC – white blood cell count, CRP – C-reactive protein, BE – base excess, FiDD – fibrin D-dimer, CK – creatine kinase, ALT – alanine aminotransferase, WSCC – water-soluble contrast challenge.

|  |  | **Nonoperative treatment failure (n=93)** | | **Nonoperative treatment success (n=209)** | **OR (95% CI)** | **p** | **Missing** |
| --- | --- | --- | --- | --- | --- | --- | --- |
| **Basic demographics** |  | |  | | | | |
| Age, median (IQR), years |  | 73 (58-83) | | 69 (58-77) | 1.01 (0.99-1.03) | 0.232 | 0 |
| Women |  | 55 (59.1) | | 101 (48.3) | 1.55 (0.94-2.54) | 0.083 | 0 |
| BMI, median (IQR), kg/m^2^ |  | 23.9 (21.6-26.2) | | 24.7 (22.6-28.9) | 0.97 (0.92-1.02) | 0.193 | 28 (9.3) |
| Comorbidities, median (IQR), Elixhauser score |  | 2 (0-3) | | 1 (1-3) | 1.06 (0.90-1.25) | 0.523 | 1 (0.3) |
| Previous abdominal operation |  | 79 (85.9) | | 186 (89.0) | 0.75 (0.36-1.56) | 0.442 | 1 (0.3) |
| Previous open abdominal operation |  | 74 (81.3) | | 173 (82.8) | 0.91 (0.48-1.71) | 0.761 | 2 (0.7) |
| Number of previous abdominal operations |  | 1 (1-3) | | 2 (1-3) | 0.89 (0.77-1.03) | 0.109 | 1 (0.3) |
| Previous pelvic surgery |  | 57 (62.0) | | 125 (60.1) | 1.08 (0.65-1.79) | 0.761 | 2 (0.7) |
| Abdominal radiotherapy |  | 4 (4.3) | | 23 (11.0) | 0.36 (0.12-1.08) | 0.069 | 53 (14.9) |
| Previous SBO |  | 20 (22) | | 92 (44) | **0.35 (0.20-0.62)** | **<0.001** | 2 (0.7) |
| Number of previous SBOs (IQR, range) |  | 0 (0; 0-10) | | 0 (0-2; 0-20) | **0.69 (0.54-0.87)** | **0.002** | 2 (0.7) |
| **History and physical** |  | |  | | | | |
| Pain severity median (IQR), range 0-10 |  | 4 (1-8) | | 4 (0-7) | 1.02 (0.95-1.10) | 0.617 | 22 (7.3) |
| Colic pain |  | 46 (50.0) | | 127 (62.0) | 0.61 (0.37-1.01) | 0.054 | 5 (1.7) |
| Constant pain |  | 46 (50.0) | | 89 (43.2) | 1.32 (0.80-2.15) | 0.277 | 4 (1.3) |
| Vomiting |  | 78 (83.9) | | 154 (74.4) | 1.79 (0.95-3.38) | 0.072 | 2 (0.7) |
| Vomiting gastric type content |  | 58 (79.5) | | 118 (69.0) | 1.74 (0.90-3.34) | 0.098 | 2 (0.7) |
| Vomiting fecal content |  | 20 (57.1) | | 36 (40.4) | 1.96 (0.89-4.33) | 0.095 | 2 (0.7) |
| Abdominal guarding (defense or peritonitis) |  | 23 (24.7) | | 35 (16.7) | 1.63 (0.90-2.96) | 0.106 | 0 |
| Bowel sounds | Normal | 21 (22.8) | | 64 (30.6) | 0.67 (0.38-1.18) | 0.168 | 1 (0.3) |
|  | Silent | 37 (40.2) | | 72 (34.4) | 1.28 (0.77-2.12) | 0.338 | 1 (0.3) |
|  | Metallic | 15 (16.3) | | 45 (21.5) | 0.71 (0.37-1.35) | 0.297 | 1 (0.3) |
|  | Splashing | 37 (40.2) | | 52 (24.9) | **2.03 (1.21-3.42)** | **0.008** | 1 (0.3) |
| MAP, mean (SD) |  | 99 (13) | | 99 (13) | 1.01 (0.99-1.02) | 0.579 | 0 |
| Pulse, median (IQR) |  | 83 (17) | | 81 (15) | 1.01 (0.99-1.03) | 0.261 | 0 |
| Temperature, median (IQR) |  | 37.0 (36.7-37.2) | | 37.0 (36.6-37.3) | 1.04 (0.65-1.67) | 0.856 | 0 |
| **Laboratory** |  | |  | | | | |
| Hb, mean (SD), g/l |  | 143 (16) | | 139 (19) | 1.01 (1.00-1.03) | 0.097 | 1 (0.3) |
| WBC, mean (IQR), E9/l |  | 10.3 (7.5-12.4) | | 9.8 (7.6-12.0) | 1.01 (0.95-1.08) | 0.683 | 0 |
| Neutrophils, median (IQR), E9/l |  | 7.8 (5.6-10.2) | | 7.4 (5.2-9.7) | 1.02 (0.95-1.09) | 0.566 | 23 (7.6) |
| Neutrophil-WBC ratio, median (IQR), % |  | 77.7 (71.6-85.3) | | 77.1 (67.2-83.4) | 1.01 (0.99-1.04) | 0.218 | 23 (7.6) |
| Thrombocytes, median (IQR), E9/l |  | 228 (194-270) | | 237 (196-282) | 1.00 (1.00-1.00) | 0.386 | 0 |
| CRP, median (IQR), mg/l |  | 15 (5-51) | | 12 (4-31) | 1.01 (1.00-1.01) | 0.083 | 0 |
| Na, median (IQR), mmol/l |  | 136 (134-139) | | 138 (136-140) | **0.94 (0.89-0.99)** | **0.029** | 0 |
| Lactate, median (IQR), mmol/l |  | 1.4 (1.1-2.0) | | 1.1 (0.9-1.6) | **1.54 (1.11-2.15)** | **0.010** | 35 (11.6) |
| pH |  | 7.40 (7.37-7.43) | | 7.39 (7.36-7.41) | 1.02 (0.96-1.08)/0.01 units | 0.543 | 44 (14.5) |
| BE, median (IQR), mmol/l |  | 2.6 (-0.1-4.8) | | 2.2 (-0.1-4.3) | 1.01 (0.94-1.09) | 0.751 | 44 (14.5) |
| FiDD, median (IQR), mg/l |  | 1.5 (.8-2.7) | | .8 (.5-1.6) | 1.07 (0.98-1.17) | 0.128 | 39 (12.9) |
| CK, median (IQR), U/l |  | 92 (51-135) | | 83 (47-137) | 1.00 (1.00-1.00) | 0.892 | 41 (13.6) |
| ALT, median (IQR), U/l |  | 20 (13-28) | | 21 (14-30) | 1.00 (0.99-1.01) | 0.610 | 11 (3.6) |
| Bilirubin, median (IQR), µmol/l |  | 14 (11-22) | | 12 (9-16) | **1.03 (1.01-1.06)** | **0.014** | 13 (4.3) |
| **Radiology** |  | |  | | | | |
| Number of transition sites, median (IQR) |  | 1 (1-2) | | 1 (1) | **2.31 (1.50-3.55)** | **<0.001** | 27 (8.9) |
| Most proximal transition site | No transition | 3 (3.3) | | 11 (5.4) |  | 0.726 | 8 (2.6) |
|  | Jejunum | 19 (20.7) | | 41 (20.3) | 1.70 (0.42-6.81) | 0.454 |  |
|  | Ileum | 68 (73.9) | | 142 (70.3) | 1.76 (0.47-6.50) | 0.399 |  |
|  | Unclear | 2 (2.2) | | 8 (4.0) | 0.92 (0.12-6.83) | 0.932 |  |
| Small bowel maximum diameter, median (IQR), mm |  | 42 (37-46) | | 40 (35-46) | 1.01 (0.98-1.04) | 0.535 | 0 |
| Closed loop sign |  | 35 (37.6) | | 17 (8.1) | **6.82 (3.56-13.1)** | **<0.001** | 0 |
| Poor bowel wall contrast enhancement |  | 4 (5.3) | | 6 (3.4) | 1.58 (0.43-5.78) | 0.487 | 49 (16.2) |
| No contrast enhancement in bowel wall |  | 1 (1.3) | | 0 (0) |  | 0.298^b^ | 50 (16.6) |
| Intense loading of enhancement in bowel wall |  | 1 (1.3) | | 4 (2.3) | 0.58 (0.06-5.25) | 0.625 | 49 (16.2) |
| Whirl sign |  | 9 (9.7) | | 19 (9.1) | 1.07 (0.46-2.45) | 0.881 | 1 (0.3) |
| Mesenteric edema or fluid |  | 79 (84.9) | | 124 (59.1) | **3.90 (2.07-7.34)** | **<0.001** | 1 (0.3) |
| Feces sign |  | 24 (25.8) | | 89 (42.6) | **0.47 (0.27-0.80)** | **0.006** | 0 |
| Peritoneal fluid |  | 55 (59.8) | | 101 (48.3) | 1.59 (0.97-2.61) | 0.068 | 1 (0.3) |
| Pneumatosis of bowel wall |  | 0 | | 0 |  |  | 2 (0.7) |
| Peritoneal gas (small bubbles) |  | 1 (1.1) | | 1 (0.5) |  | 0.520^b^ | 2 (0.7) |
| WSCC done |  | 70 (75.3) | | 175 (84.1) | 0.57 (0.32-1.05) | 0.070 | 1 (0.3) |
| Nasogastric tube outflow 12h |  | 815 (285-1600) | | 489 (129-1091) | 1.01 (0.99-1.04)/100ml | 0.198 | 41 (13.6) |

**Table S4** Variable selection to the nonoperative treatment failure (NOFA) prediction model. In addition, STIRSK model variable selection was used for NOFA model development. Finally, the same variables were used for both models. Marking with “x” if determined important by univariable analysis, literature (variable associated with nonoperative treatment failure in previous literature) or by clinical assessment. Marked “(x)” if potentially difficult to use clinically. Mesenteric edema or fluid and peritoneal fluid are included as combined variable.

SBO – small bowel obstruction WBC- white blood cell count, FiDD – fibrin D-dimer, CK – creatine kinase, NG tube – nasogastric tube.

| **Variable** | **Univariable analysis suggests** | **Literature suggests** | **Clinically useful** | **Argument against use in prediction model** | **Literature reference** |
| --- | --- | --- | --- | --- | --- |
| Previous SBO | x | x | x |  | (18, 19) |
| **Number of previous SBOs** | x | x | x |  | (3, 19) |
| Charlson index |  | x |  | Slow to assess at ED | (20) |
| **Abdominal guarding** |  |  | x |  |  |
| Splashing bowel sounds | x |  | (x) | Subjective |  |
| Neutrophils | x |  | x |  |  |
| **Neutrophil-WBC -ratio** |  |  | x |  |  |
| Na | x |  | (x) | Affected by numerous medical conditions |  |
| Lactate | x |  | (x) | May not be routinely in use |  |
| FiDD | x |  | (x) | May not be routinely in use |  |
| Bilirubin | x |  | (x) | May not be routinely in use |  |
| CK |  | x | (x) | May not be routinely in use | (21) |
| NG tube outflow | x |  | x |  |  |
| Transition site location |  | x | (x) | May be hard to detect or report | (22) |
| Distal obstruction |  | x | x |  | (20) |
| Number of transition sites | x |  | x |  |  |
| **Closed loop sign** | x | x | x |  | (18, 23) |
| **Mesenteric edema or fluid** | x | x | x |  | (18, 23, 24) |
| **Peritoneal fluid** |  | x | x |  | (19, 23) |
| **Feces sign** | x | x | x |  | (4, 24) |
| Small bowel maximum diameter |  | x | x |  | (19, 20) |
| Beak sign |  | x | x |  | (19) |
| Number of beak signs |  | x | x |  | (22) |

**Figure S1** Continuous predictors’ association with the logit of strangulation (left) and nonoperative treatment failure (right) using restricted cubic splines. In model development, NL-ratio was modelled as linear term as using restricted cubic splines did not differ significantly.

**Figure S2** The strangulation model regression coefficient demonstrated inadequate calibration (intercept = 1.821, slope = 0.746) for predicting nonoperative treatment failure. Predicted probability on x-axis, actual probability on y-axis, perfectly calibrated prediction model would have a diagonal calibration curve (reference line). Histograms represent the distribution of predicted risk.

**Figure S3** ROC-curves of strangulation model (left) and nonoperative treatment failure model (right). Cutoff-points for higher specificity or higher sensitivity and their positive and negative predictive values (PPV and NPV) are shown.

**References**

1. Bouassida M, Laamiri G, Zribi S, Slama H, Mroua B, Sassi S, et al. Predicting Intestinal Ischaemia in Patients with Adhesive Small Bowel Obstruction: A Simple Score. World J Surg. 2020;44(5):1444-1449.

2. Wassmer CH, Revol R, Uhe I, Chevallay M, Toso C, Gervaz P, et al. A new clinical severity score for the management of acute small bowel obstruction in predicting bowel ischemia: a cohort study. Int J Surg. 2023;109(6):1620-1628.

3. Behman R, Nathens AB, Mason S, Byrne JP, Hong NL, Pechlivanoglou P, et al. Association of Surgical Intervention for Adhesive Small-Bowel Obstruction With the Risk of Recurrence. JAMA Surg. 2019;154(5):413-420.

4. Zielinski MD, Eiken PW, Bannon MP, Heller SF, Lohse CM, Huebner M, et al. Small bowel obstruction-who needs an operation? A multivariate prediction model. World J Surg. 2010;34(5):910-919.

5. Schwenter F, Poletti PA, Platon A, Perneger T, Morel P, Gervaz P. Clinicoradiological score for predicting the risk of strangulated small bowel obstruction. Br J Surg. 2010;97(7):1119-1125.

6. Mu JF, Wang Q, Wang SD, Wang C, Song JX, Jiang J, et al. Clinical factors associated with intestinal strangulating obstruction and recurrence in adhesive small bowel obstruction: A retrospective study of 288 cases. Medicine (Baltimore). 2018;97(34):e12011.

7. Duron JJ, Silva NJ, du Montcel ST, Berger A, Muscari F, Hennet H, et al. Adhesive postoperative small bowel obstruction: incidence and risk factors of recurrence after surgical treatment: a multicenter prospective study. Ann Surg. 2006;244(5):750-757.

8. Jancelewicz T, Vu LT, Shawo AE, Yeh B, Gasper WJ, Harris HW. Predicting strangulated small bowel obstruction: an old problem revisited. J Gastrointest Surg. 2009;13(1):93-99.

9. Woodford EP, Woodford HM, Hort AR, Pang TC, Lam VWT, Nahm CB. Neutrophil-lymphocyte ratio and platelet-lymphocyte ratio use in detecting bowel ischaemia in adhesional small bowel obstruction. ANZ J Surg. 2022;92(11):2915-2920.

10. Cosse C, Regimbeau JM, Fuks D, Mauvais F, Scotte M. Serum procalcitonin for predicting the failure of conservative management and the need for bowel resection in patients with small bowel obstruction. J Am Coll Surg. 2013;216(5):997-1004.

11. O'Leary MP, Neville, A. L., Keeley, J. A., Kim, D. Y., de Virgilio, C., Plurad D. S. Predictors of Ischemic Bowel in Patients with Small Bowel Obstruction. The American Surgeon. 2016;82(10):992-994.

12. Millet I, Boutot D, Faget C, Pages-Bouic E, Molinari N, Zins M, et al. Assessment of Strangulation in Adhesive Small Bowel Obstruction on the Basis of Combined CT Findings: Implications for Clinical Care. Radiology. 2017;285(3):798-808.

13. Zielinski MD, Eiken PW, Heller SF, Lohse CM, Huebner M, Sarr MG, et al. Prospective, observational validation of a multivariate small-bowel obstruction model to predict the need for operative intervention. J Am Coll Surg. 2011;212(6):1068-1076.

14. Morris RS, Murphy P, Boyle K, Somberg L, Webb T, Milia D, et al. Bowel Ischemia Score Predicts Early Operation in Patients With Adhesive Small Bowel Obstruction. Am Surg. 2022;88(2):205-211.

15. Millet I, Taourel P, Ruyer A, Molinari N. Value of CT findings to predict surgical ischemia in small bowel obstruction: A systematic review and meta-analysis. Eur Radiol. 2015;25(6):1823-1835.

16. Chang WC, Ko KH, Lin CS, Hsu HH, Tsai SH, Fan HL, et al. Features on MDCT that predict surgery in patients with adhesive-related small bowel obstruction. PLoS One. 2014;9(2):e89804.

17. Matsushima K, Inaba K, Dollbaum R, Cheng V, Khan M, Herr K, et al. High-Density Free Fluid on Computed Tomography: a Predictor of Surgical Intervention in Patients with Adhesive Small Bowel Obstruction. J Gastrointest Surg. 2016;20(11):1861-1866.

18. Demessence R, Lyoubi Y, Feuerstoss F, Hamy A, Aube C, Paisant A, et al. Surgical management of adhesive small bowel obstruction: Is it still mandatory to wait? - An update. J Visc Surg. 2022;159(4):309-319.

19. Paisant A, Burgmaier J, Calame P, Loison M, Moliere S, Brigand C, et al. The Angers CT Score is a Risk Factor for the Failure of the Conservative Management of Adhesive Small Bowel Obstruction: A Prospective Observational Multicentric Study. World J Surg. 2023;47(4):975-984.

20. Maraux L, Dammaro C, Gaillard M, Lainas P, Derienne J, Maitre S, et al. Predicting the Need for Surgery in Uncomplicated Adhesive Small Bowel Obstruction: A Scoring Tool. J Surg Res. 2022;279:33-41.

21. Tanaka S, Yamamoto T, Kubota D, Matsuyama M, Uenishi T, Kubo S, et al. Predictive factors for surgical indication in adhesive small bowel obstruction. Am J Surg. 2008;196(1):23-27.

22. Millet I, Ruyer A, Alili C, Doyon F, Molinari N, Pages E, et al. Adhesive small-Bowel Obstruction: Value of CT in Identifying Findings Associated with the Effectiveness of Nonsurgical Treatment. Radiology. 2014;273(2):425-432.

23. Eze VN, Parry T, Boone D, Mallett S, Halligan S. Prognostic factors to identify resolution of small bowel obstruction without need for operative management: systematic review. Eur Radiol. 2023;34:3861–3871.

24. Kim J, Lee Y, Yoon JH, Lee HJ, Lim YJ, Yi J, et al. Non-strangulated adhesive small bowel obstruction: CT findings predicting outcome of conservative treatment. Eur Radiol. 2021;31(3):1597-1607.
